# Lower Serum Uric Acid Is Associated With Post-Stroke Depression at Discharge

**DOI:** 10.3389/fpsyt.2020.00052

**Published:** 2020-02-18

**Authors:** Guo Li, Jinfeng Miao, Wenzhe Sun, Xiaoyan Song, Yan Lan, Xin Zhao, Xiuli Qiu, Chenyan Zhang, Zhou Zhu, Suiqiang Zhu

**Affiliations:** Department of Neurology, Tongji Hospital, Tongji Medical College, Huazhong University of Science and Technology, Wuhan, China

**Keywords:** post-stroke depression, serum uric acid, restricted cubic spline regression, antioxidants, threshold effect

## Abstract

**Background:**

Serum uric acid (SUA) has been shown to play an important role in the pathophysiology of mood disorders including 3- and 6-month post-stroke depression (PSD). This study aimed to investigate whether SUA levels on admission were associated with PSD at discharge.

**Methods:**

A total of 498 stroke patients were consecutively recruited from Tongji Hospital. Clinical and laboratory test data were collected on admission. They were categorized into equal tertiles according to the distribution of SUA and the number of patients. PSD status was evaluated by DSM-V criteria and 17-item Hamilton Rating Scale for Depression at discharge.

**Results:**

The optimal cut-off points of SUA were: (T1) 80.00~300.80 µmo1/L, (T2) 300.81~391.67 µmo1/L, (T3) 391.68~710.0 µmo1/L. A total of 232 patients (46.59%) were diagnosed as PSD at discharge. Significant differences were found between the PSD and non-PSD groups in SUA tertiles of patients (*P* = 0.00). After adjustment for conventional confounding factors, the odds ratios of PSD were 5.777 (95% CI = 3.463~9.637, *P* = 0.00) for the lowest tertile and 4.153 (95% CI = 2.492~6.921, *P* = 0.00) for the middle tertile of SUA, as compared with the highest tertile. In restricted cubic spline regression, continuous SUA showed linear relation with PSD risk at discharge after 300 µmol/L.

**Conclusions:**

Lower SUA levels on admission were found to be associated with PSD at discharge and the threshold effect was also revealed. For stroke patients, doctors should pay attention to the baseline SUA for screening high-risk PSD at discharge in clinical practice.

## Introduction

Post-stroke depression (PSD) is the most frequent neuropsychiatric sequela after stroke, affecting about 40% of stroke patients (1, 2). It is well known that PSD was associated with reduced quality of life (QoL), poorer functional outcome, as well as increased cost of treatment, burden of family caregiver, and mortality (1, 3–5). Moreover, a recent meta-analysis reported that patients with early PSD had a mortality risk about 1.5 higher than non-depressed individuals, considering both short- and long-term mortality (6). The depression effects on cardiovascular risk, such as unhealthy lifestyle behaviors and lower adherence to treatment, the higher stroke severity, possible negative effects of a SSRI treatment on survival, and non-natural causes of death like suicide may be the potential interpretations (6). Although the importance of PSD has been well documented and there are validated screening tools for PSD, many PSD patients cannot be diagnosed and rate of refusal by busy stroke clinicians to recommend antidepressant treatment remain high. One reason is that there are no reliable objective biomarkers for diagnosing and predicting PSD.

To date, there is a lack of understanding of what the exactly underlying pathophysiological mechanisms of PSD are (7). Among these discovered biological factors, oxidative and nitrosative stress pathways are regarded as the most conclusive factors because there are low antioxidant levels and high metabolic rates and levels in the brain (8, 9). As we know, the brain is a site of excessive reactive oxygen species (ROS) production and especially vulnerable to oxidative stress, as it accounts for 20% oxygen consumed by the body (10). Hence, brain neurons damaged by oxidate stress may lead to altered membrane structure and function, which may further affect the expression of membrane receptors leading to increased risk of depression (11, 12). Serum uric acid (SUA) is an important antioxidant which is the end product in the degradation of the purine nucleotides adenine and guanine, accounting approximately 60% of the total antioxidant capacity in plasma (13). Meanwhile, SUA is a low-cost indicator which can be easily obtained on admission. A recent meta-analysis has shown that subjects with major depressive disorders (MDD) have levels of the antioxidate uric acid (UA) lower than healthy controls (14). Moreover, previous studies have shown that the lower SUA level is associated with both PSD and depression (9, 15). However, the assessment on the association between SUA and PSD only aimed at 3- and 6-month outcomes. It is still lacking objective and quantitative biological factors to assess PSD at discharge. When the early onset of PSD is ignored by clinicians, it will adversely affect the early rehabilitation after discharge, which is the most important stage for stroke rehabilitation. Thus, we need effective biological predictors in order to more efficiently identify and diagnose PSD at discharge, and then intervene to promote early recovery of stroke.

Therefore, considering that SUA is proven to be an effective predictor of 3- and 6-month PSD, we hypothesized that the SUA may also serve as a predictor of patients with PSD at discharge. The aim of this study was to assess the association between SUA on admission and the PSD outcome at discharge.

## Methods

### Study Design

All first-ever stroke patients were consecutively recruited within 7 days of the onset of symptoms from the Tongji Hospital which is located in Wuhan City, Hubei Province, China, between May 2018 and October 2019. SUA was obtained within 24 h after admission. There are few clinical concerns about the decreased odds ratio (OR) value in PSD for each unit change of UA, which could be obtained by taking SUA as a continuous measure. Therefore, we divided the SUA into tertiles to observe whether any enhanced performance could be quantified while maintaining statistical effect in each category, according to the patients' amount and the skewed distribution of the raw SUA values (7, 16). All patients involved in this study or their family members gave written informed consents according to the Declaration of Helsinki. The approval of the study for experiments was obtained from the Ethics Committee of Tongji Medical College, Huazhong University of Science and Technology.

The registration number of this prospective cohort study was ChiCTR-ROC-17013993. The URL of the publicly accessible website on which this trial is registered is: http://www.chictr.org.cn/index.aspx. The original protocols used for this *post hoc* analysis did not include SUA as a potential predictor.

### Inclusion and Exclusion Criteria

All suspected stroke patients were confirmed by magnetic resonance imaging or computerized tomography within 7 days after admission and the following inclusive criteria were used: (i) males and females, age ≥ 18 years old, first-ever diagnosed stroke patients, including ischemic and hemorrhagic stroke; (ii) hospitalized within 7 days after stroke onset; (iii) written informed consent was provided.

The exclusive criteria were as follows: (i) brain dysfunction caused by other non-vascular causes, such as primary brain tumors, subdural hematoma, paralysis after seizures, metastatic encephaloma, brain trauma, etc.; (ii) history of depression (previous treatment history or clinical diagnosis), dementia and/or other psychiatric illness; (iii) communication problems due to aphasia, dysarthria, disturbance of understanding or consciousness (a Mini-Mental State Examination score was <19 points, in particular the MMSE score of illiterate patients was <17 points); (iv) unable to complete the follow up; (v) Transient ischemic attack and subarachnoid hemorrhage; (vi) with other concomitant neuropsychiatric diseases, such as Parkinson's disease and epilepsy.

Serum samples were collected at room temperature on admission, then centrifuged at 3,500 r/min for 10 min, which could be used to measure the levels of serum biomarkers. Depressive symptoms were measured at discharge, while the baseline sociodemographic information, clinical characteristics, and routine laboratory indicators were collected on admission.

### Data Collection and Follow-Up

Standard patient demographic data was collected with a case report form at baseline, covering gender, age, body mass index (BMI), marital status, degree of education, and vascular risk factors, including smoking, drinking, and history of stroke, diabetes mellitus, hypertension, hyperlipidemia, coronary heart disease, and surgery. The stroke severity was assessed within 24 h of hospital admission by well-trained doctors using the National Institutes of Health Stroke Scale (NIHSS) score. Barthel Index (BI) score, mRS score, and hospitalization days were also included into the variables. The concentrations of SUA, serum albumin (ALB), homocysteine (Hcy), hypersensitive C-reactive protein (Hs-CRP) were measured by standard autoanalyzer techniques with a Roche automatic analyzer (cobas c 701) in clinical lab of Tongji Hospital. The inflammatory factors, including IL-1β, IL-6, IL-10, IL-18, TNF-α, BDNF, and IFN-γ, were measured using a solid-phase sandwich enzyme-linked immunosorbent assay kit (CUSABIO, China) according to the manufacturer's specifications in Kindstar Company, Wuhan. To minimize assay variance, all samples were analyzed on the same day in duplicate in a random order by a technician blind to the clinical diagnoses; the intra-assay coefficients were <5%.

### Psychological Measurement

All psychological evaluations were performed by two experienced psychiatrists (X.S. and W.S.) who were blinded to other clinical and laboratory findings after receiving standardized training. The interrater reliability reached an acceptable level. PSD was diagnosed by a psychiatrist at discharge according to DSM-V criteria. Seventeen-item Hamilton Rating Scale for Depression (HRSD) was used to measure the degree of PSD at discharge. The DSM-V diagnostic criteria (Depressive Disorder Due to Another Medical Condition) was met, and HRSD score≥7 at discharge, which was regarded as the primary endpoint. Patients were divided into PSD group and non-PSD group according to whether they had PSD outcome or not. The validity and reliability of the Chinese HRSD version had been proven in previous studies (17).

### Statistical Analysis

Results were expressed as percentages for categorical variables and as medians [interquartile range (IQR)] or means ± standard deviation (S.D.) for the continuous variables, depending on the normal or nonnormal distribution of data by Kolmogorov-Smirnov test. Proportions were compared using the Chi-squared test, Student's t-test, and analysis of variance (ANOVA) were employed for the normally distributed variables, while the Mann–Whitney U-test was used for the asymmetrically distributed variables. Statistical comparisons among SUA stratification were assessed by Pearson's Chi-square test or Fisher's exact test for categorical variables, and continuous variables were evaluated by Kruskal–Wallis test or ANOVA. After adjusting for main baseline variables identified in the univariate logistic regression analysis and traditional confounders related to PSD, the OR values and 95% confidence intervals (95% CIs) for PSD risk were obtained by multivariate-adjusted binary logistic regression. The restricted cubic spline (RCS) regression was used to test the linear association between PSD and SUA as a continuous measure with three knots.

All statistical analysis was performed with R version 3.5.2 and SPSS for Windows, version 22.0 (SPSS Inc., Chicago, IL, USA). Statistical significance was identified as a two-tailed *P* values less than 0.05 (*P* < 0.05). The R packages “rms”, “Hmisc”, “Formula” and “ggplot2” were applied.

## Results

### Baseline Characteristics of all Patients in SUA Tertiles

A total of 707 stroke patients from Tongji Hospital between May 2018 and October 2019 were recruited in the study, and 567 patients were eligible for the research. By the time of discharge, there were 69 patients failed to be followed up. Ultimately, we included a total of 498 stroke patients, which consisted of 379 males (76.10%) and 119 females (23.90%). Their mean age was 57.17 ± 10.88 years (**Table 1**). We divided all cases into three groups according to tertiles of SUA levels, which ensured the most categories with adequate number of patients per subgroups between the range of 80.00 and 710.00 µmo1/L (T1, 166 patients; T2, 166 patients; T3, 166 patients).

**Table 1:** Baseline characteristics of patients with stroke according to SUA tertiles.

Variables	All patients	SUA tertiles			*P*-value
		Tertile 1, n = 166 (80.0~300.80)	Tertile 2, n = 166 (300.81~391.67)	Tertile 3, n = 166 (391.68~710.0)	
UA, median (IQR)	328.0 (275.7, 410.00)	253.00 (215.25, 276.00)	328.00(328.00, 361.63)	410.00 (410.00, 448.00)	0.000
**Demographic parameters**					
Age (years)	57.17 ± 10.88	58.25 ± 9.88	58.01 ± 11.34	55.23 ± 11.17	0.019
Females, n (%)	119 (23.90)	57 (34.34)	34 (20.48)	28 (16.87)	0.000
BMI (kg/m^2^)	24.96 ± 3.54	24.25 ± 3.27	25.05 ± 3.69	25.59 ± 3.53	0.002
Married, n (%)	483 (96.99)	161 (96.99)	160 (96.39)	162 (97.59)	0.814
Education level					0.111
Junior middle school and below, n (%)	306 (61.45)	114 (68.67)	99 (59.64)	93 (56.02)	0.051
Senior high/polytechnic school, n (%)	126 (25.30)	38 (22.89)	42 (25.30)	46 (27.72)	0.601
Bachelor and above, n (%)	66 (13.25)	14 (8.43)	25 (15.06)	27 (16.27)	0.077
**Vascular risk factors**					
Smoking, n (%)	297 (59.64)	79 (47.59)	96 (57.83)	122 (73.49)	0.000
Drinking, n (%)	302 (60.64)	87 (52.41)	104 (62.65)	111 (66.87)	0.021
History of diabetes, n (%)	106 (21.29)	45 (21.11)	41 (24.70)	20 (12.05)	0.002
History of hypertension, n (%)	273 (54.82)	88 (53.01)	103 (62.05)	82 (49.40)	0.058
History of hyperlipidemia, n (%)	109 (21.89)	27 (16.27)	45 (27.11)	37 (22.29)	0.057
Coronary artery diseases, n (%)	51 (10.24)	17 (10.24)	19 (11.45)	15 (9.04)	0.769
History of previous stroke, n (%)	92 (18.47)	25 (15.06)	32 (19.28)	35 (21.08)	0.349
History of surgery, n (%)	158 (31.73)	61 (36.75)	54 (32.53)	43 (25.90)	0.101
**Clinical characteristics**					
NIHSS score, median (IQR)	3 (2, 6.5)	4 (2, 8)	4 (2, 7)	3 (1, 5)	0.001
BI score, median (IQR)	85 (45, 100)	72.5 (40, 100)	80 (48.75, 100)	95 (65, 100)	0.000
mRS score, median (IQR)	2 (1, 4)	3 (2, 4)	3 (1,4)	2 (1, 3)	0.000
HRSD score, median (IQR)	7 (4, 12)	9 (5,13.25)	9.0 (4.75, 13.00)	5 (3, 7)	0.000
Hospitalization days, median (IQR)	9 (7, 16)	9 (7,14)	10 (7, 16)	9.5 (6, 16)	0.926
**Serum biochemicals**					
Albumin, median (IQR)	41.07 (39.60, 42.60)	40.65 (38.18, 42.63)	41.07 (40.10, 42.80)	41.07 (41.07, 42.13)	0.013
Hcy, median (IQR)	15.10 (11.70, 15.3)	12.60 (10.30, 15.10)	15.10 (12.15, 15.10)	15.10 (14.50, 16.95)	0.000
Hs-CRP, median (IQR)	3.85 (1.10, 8.26)	2 (0.60, 8.26)	3.45 (1.10, 8.26)	8.26 (1.98, 8.26)	0.000
IL-1β, median (IQR)	61.14 (18.62, 135.82)	50.43 (14.27, 135.82)	50.51 (18.77, 135.82)	119.01 (28.25,163.83)	0.003
IL-6, median (IQR)	4.48 (1.93, 9.47)	3.63 (1.86, 9.30)	5.03 (2.08, 9.47)	4.75 (1.88, 9.47)	0.730
IL-10, median (IQR)	8.72 (2.30, 23.46)	8.08 (2.10,18.17)	9.26 (2.27, 23.80)	11.26 (2.48, 29.68)	0.268
IL-18, median (IQR)	1,773.41 (568.82, 3,520.77)	1,503.89 (397.79,3,520.77)	1,641.50 (575.27, 3,520.77)	2,552.29 (780.13, 3,534.55)	0.043
TNF-α, median (IQR)	36.73 (18.19, 49.53)	36.75 (19.32, 51.51)	38.53 (19.10, 54.59)	33.32 (15.33, 43.33)	0.062
BDNF, median (IQR)	3.60 (1.96, 5.78)	3.21 (1.63, 5.92)	3.67 (2.18, 5.72)	3.86 (2.14, 6.13)	0.251
IFN-γ, median (IQR)	4.43 (1.73, 9.72)	4.50 (1.54, 8.99)	4.41 (1.83, 8.40)	4.32 (1.71, 9.79)	1.000

BMI, body mass index; BI, Barthel Index; mRS, modified Rankin Scale; NIHSS, National Institutes of Health Stroke Scale; SUA, serum uric acid; Hcy, homocysteine;

Hs-CRP, hyper-sensitive C-reactive protein; TNF, tumor necrosis factor; BDNF, brain derived neurotrophic factor; IFN, interferon; IL, interleukin.

The cut-off values for this stratification on the SUA level into tertiles were: (T1) 80.00~300.80 µmo1/L, (T2) 300.81~391.67 µmo1/L, (T3) 391.68~710.0. **Table 1** summarized the characteristics of the patients by the tertiles of SUA, including the sociodemographic, clinical, and laboratory characteristics. Patients with the low, moderate, and high SUA were significantly different in following sociodemographic and clinical variables: age, gender, BMI, smoking, drinking, history of diabetes and hypertension, HRSD score at discharge, baseline NIHSS score, BI score, and mRS score. The majority of patients had drinking (60.64%) and smoking habits (59.64%) and the lowest and middle SUA tertiles were significantly associated with higher HRSD score than the highest tertile (*P* = 0.00). And there were statistical differences observed for ALB, Hcy, Hs-CRP, IL-1β, and IL-18 in the SUA tertiles of patients (all *P* values < 0.05). Some significant differences in baseline characteristics were reasonable because the patients were consecutively recruited.

### Baseline Characteristics of Patients in PSD Group and Non-PSD Group

The baseline characteristics between PSD and non-PSD groups are presented in **Table 2**. In this study, 232 (46.59%) patients were diagnosed as PSD at discharge. Compared with non-PSD group, PSD patients were more likely to be older and with lower educational level and BI scores, higher proportion of coronary artery disease history, surgery history, HRSD scores, baseline NIHSS, and mRS scores.

**Table 2:** Clinical and demographic characteristics of patients with PSD and non-PSD.

Variables	PSD patients (n = 232)	Non-PSD patients (n = 266)	*P*-value
SUA	328 (266.08, 348.45)	389 (295.5, 410)	0.000
**Demographic parameters**			
Age (years)	58.34 ± 10.49	56.14 ± 11.13	0.024
Females, n (%)	59 (25.43)	60 (22.56)	0.453
BMI (kg/m^2^)	24.83 ± 3.26	25.08 ± 3.76	0.426
Married, n (%)	225 (96.98)	258 (96.99)	0.995
Education level			0.018
Junior middle school and below, n (%)	155 (66.81)	151 (56.77)	
Senior high/Polytechnic school, n (%)	56 (24.14)	70 (26.32)	
Bachelor/Junior college and above, n (%)	21 (9.05)	45 (16.92)	
**Vascular risk factors**			
Smoking, n (%)	133 (57.33)	164 (61.65)	0.326
Drinking, n (%)	139 (59.91)	163 (61.28)	0.756
History of diabetes, n (%)	52 (22.41)	54 (20.30)	0.566
History of hypertension, n (%)	131 (56.47)	142 (53.38)	0.491
History of hyperlipidemia, n (%)	52 (22.41)	57 (21.43)	0.791
Coronary artery diseases, n (%)	31 (13.36)	20 (7.52)	0.032
History of previous stroke, n (%)	46 (19.83)	46 (17.29)	0.467
History of previous surgery, n (%)	84 (36.21)	74 (27.82)	0.045
**Clinical characteristics**			
NIHSS score, median (IQR)	5 (2, 8)	2 (1, 4)	0.000
BI score, median (IQR)	65 (35, 95)	95 (68.75, 100)	0.000
mRS score, median (IQR)	3 (2, 4)	2 (1, 3)	0.000
HRSD score, median (IQR)	12 (10, 16)	4 (2, 6)	0.000
Hospitalization days, median (IQR)	10 (7, 15)	9 (6, 16)	0.486
**Serum biochemicals**			
Albumin, median (IQR)	41.07 (39.20, 42.48)	41.07 (40.30, 42.80)	0.140
Homocysteine, median (IQR)	14.65 (11.73, 15.10)	15.10 (11.70, 15.40)	0.353
Hs-CRP, median (IQR)	3.50 (1.10, 8.26)	4.45 (1.10, 8.26)	0.712
IL-1β, median (IQR)	55.51 (18.90, 135.82)	67.32 (18.19, 135.91)	0.467
IL-6, median (IQR)	4.75 (1.84, 9.47)	4.28 (1.97, 9.47)	0.377
IL-10, median (IQR)	8.15 (1.95, 23.24)	9.50 (2.50, 23.64)	0.620
IL-18, median (IQR)	1,681.96 (503.52, 3,520.77)	1,938.46 (680.12, 3,520.77)	0.496
TNF-α, median (IQR)	36.84 (18.32, 50.95)	36.04 (17.66, 48.50)	0.710
BDNF, median (IQR)	3.34 (1.85, 5.75)	3.80 (2.12, 5.93)	0.393
IFN-γ, median (IQR)	4.56 (1.84, 9.73)	4.21 (1.71, 8.71)	0.357

BMI, body mass index; BI, Barthel Index; mRS, modified Rankin Scale; NIHSS, National Institutes of Health Stroke Scale; SUA, serum uric acid; Hcy, homocysteine; Hs-CRP, hyper-sensitive C-reactive protein; TNF, tumor necrosis factor; BDNF, brain-derived neurotrophic factor; IFN, interferon; IL, interleukin.

We also divided the SUA levels into hyperuricemic and non-hyperuricemic groups, in which hyperuricemia is defined as 416.5 µmol/L (7.0 mg/dl) or higher in males, and 357 µmol/L (6.0 mg/dl) or higher in females. The association between hyperuricemic and PSD was explored by univariate and multivariate adjusted logistic regression models. We could speculate that hyperuricemia might protect from PSD from the regression results (OR ≈ 0.42, 95% CI ≈ 0.26~0.69, *P* = 0.001).

Moreover, we considered the ALB level as the representative index of overall nutritional status. There were differences in ALB levels across the SUA tertiles, as shown in **Table 1**. The *post hoc* test after Mann-Whitney U test declared that the ALB in lowest tertile SUA group was significantly less than the middle and the highest tertile SUA groups (both *P* = 0.032). Moreover, there was no difference in ALB levels between PSD and non-PSD groups (*P* = 0.140, **Table 2**). The subjects in the lowest tertile SUA group may be with worse nutritional status and decreased food intake. It reminds that there may be more risk factors, such as unhealthy lifestyles rather than ALB itself, leading to PSD prior to admission in the lowest SUA tertile group.

### Association Between the Level of SUA and PSD

Significant differences were found between the PSD and non-PSD groups in SUA tertiles of patients (all *P* < 0.05, **Table 3**). Indeed, the proportions of patients in the lowest tertile (80.00~300.80 µmo1/L, *P* = 0.001) and the middle tertile (300.81~391.67 µmo1/L, *P* = 0.00) were significantly higher in the PSD groups, whilst the proportion of patients in the highest tertile (391.68~710.00 µmo1/L) was significantly lower in the PSD group (*P* = 0.00) (**Table 3**). In **Table 4**, with all patients taken as a whole, PSD occurrence taken as a dependent variable and highest tertile taken as the reference was used for SUA in the unadjusted and multivariate adjusted logistic regression models. In unadjusted logistic regression model, the lowest tertile of SUA was independently consistent with a risk predictor of PSD with an unadjusted OR of 5.965 (95% CI = 3.667~9.704, *P* = 0.00). The middle tertile of SUA was independently consistent with a risk predictor of PSD with an unadjusted OR of 5.008 (95% CI = 3.089~8.120, *P* = 0.00).

**Table 3:** SUA tertiles of patients.

Variable	PSD patients (n = 232)	Non-PSD patients (n = 266)	χ^2^	*P*-value
SUA			65.672	0.000
Tertile 1, n = 166 (80~300.8 µmol/L)	95	71	11.334	0.001
Tertile 2, n = 166 (300.81~391.67 µmol/L)	102	64	22.095	0.000
Tertile 3, n = 166 (391.68~710 µmol/L)	35	131	65.078	0.000

SUA, serum uric acid; PSD, post-stroke depression.

**Table 4:** Multivariate adjusted odds ratios for the association between SUA levels and PSD at discharge.

	Tertile	OR^a^	95% CI	*P*-value
Unadjusted	Lowest	5.965	3.667~9.704	0.00
	Moderate	5.008	3.089~8.120	0.00
Model 1^b^	Lowest	6.009	3.682~9.808	0.00
	Moderate	4.776	2.937~7.767	0.00
Model 2^c^	Lowest	6.009	3.674~9.829	0.00
	Moderate	4.808	2.949~7.838	0.00
Model 3^d^	Lowest	5.777	3.463~9.637	0.00
	Moderate	4.153	2.492~6.921	0.00

OR, odds radio; CI, confidence level; PSD, post-stroke depression.

^a^Reference OR (1.000) is the highest tertile of SUA for PSD.

^b^Model 1: adjusted for age, gender, education levels, body mass index, smoking, drinking.

^c^Model 2: adjusted for covariates from model 1 and further adjusted for medical history (coronary artery disease, diabetes mellitus, hyperlipidemia, hypertension, previous stroke, and surgery).

^d^Model 3: adjusted for covariates from model 2 and further adjusted for baseline NIHSS scores, mRS scores, and Barthel Index scores.

After adjusting for conventional confounders including age, gender, education levels, BMI, smoking, drinking, history of coronary artery disease, diabetes mellitus, hyperlipidemia, hypertension, previous stroke and surgery, baseline NIHSS, mRS and BI scores, and the lowest tertile of SUA was remained significant independently associated with the prevalence of PSD (model 1: OR = 6.009, 95% CI = 3.682~9.808, *P* = 0.00; model 2: OR = 6.009, 95% CI = 3.674~9.829, *P* = 0.00; model 3: OR = 5.777, 95% CI = 3.463~9.637, *P* = 0.00), as compared with the highest tertile. Similarly, the middle tertile of SUA was remained significant independently associated with the prevalence of PSD (model 1: OR = 4.776, 95% CI = 2.937~7.767, *P* = 0.00; model 2: OR = 4.808, 95% CI = 2.949~7.838, *P* = 0.00; model 3: OR = 4.153, 95% CI = 2.492~6.921, *P* = 0.00).

**Figure 1:**
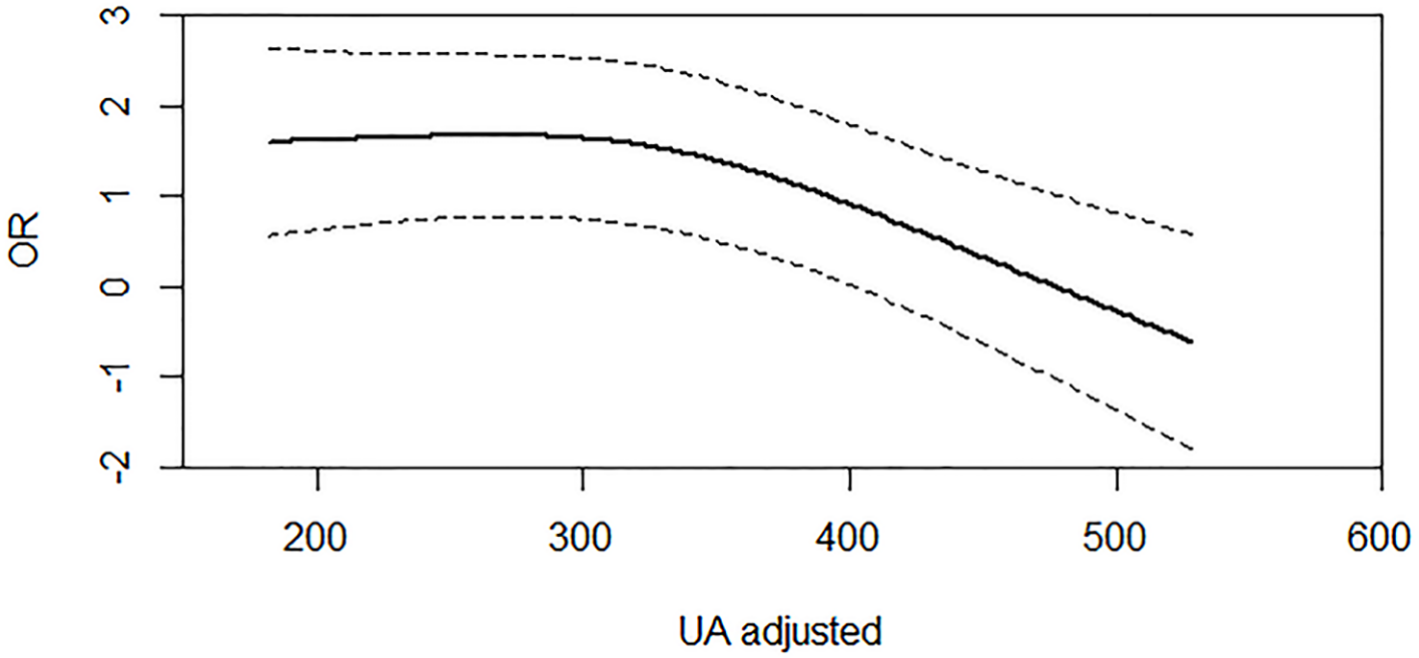
Association of serum UA (uric acid) levels with risk of PSD. Dashed lines are 95% confidence intervals. Odds ratios and 95% confidence intervals derived from restricted cubic spline regression (*P* for nonlinear = 0.0119). Odds ratios were estimated using logistic regression modeling, adjusting for the same variables as model 3 in **Table 4**.

Furthermore, we used RCS regression model to confirm the linear relationship between continuous SUA levels and PSD at discharge (χ^2^ = 6.33, df = 2, *P* = 0.0119 for nonlinearity, **Figure 1**). The RCS curve showed that there was nonlinear effect between SUA and PSD outcome. However, the linear association was found between SUA and PSD after the value of around 300 µmol/L. Coincidentally, the cut-off point of RCS curve (300 µmol/L) was approximately the same as the first tertile split points (300.8 µmol/L). It is a reasonable approach to take the tertiles of SUA levels to analyze the association between SUA and PSD risk. There is a protective effect in PSD when the SUA level was more than 480 µmo1/L, while the 95% CI of other SUA values (≤480 µmo1/L) is not completely above or below the value “1” of significance.

## Discussion

In this study, we have investigated the association between SUA levels on admission and the development of PSD outcome at discharge using a prospective cohort. Our results suggested that lower levels of SUA on admission were associated with the presence of PSD at discharge, and documented that the risk of PSD in patients significantly declined with the increasing SUA level after 300 µmol/L. Therefore, our findings revealed the SUA value could provide important information for predicting PSD patients at discharge. A total of 46.59% of stroke patients presented with PSD in this study, which is more than the incidence reported in previous studies. This could be potentially explained by that Tongji hospital was the highest-ranked large general 3A hospital in Central China and recruited more serious stroke patients whose the median NIHSS score (IQR) was 3 (2, 6.5) (7). Previous study has reported the higher levels of SUA on admission was associated with the occurrence of 3-month major PSD, while lower level of SUA on admission was closely related to the occurrence of major PSD between 3- and 6-month post-stroke. Six months after acute ischemic stroke, there was no relationship between major PSD and baseline SUA (15). The differences in time of assessment and diagnosis, subjects' samples, and psychiatric assessment methods could explain the discrepancies between our study and their findings.

Compared with the patients without PSD at discharge, our results also demonstrated that the PSD patients had significantly increased stroke severity (higher NIHSS scores) and worse functional outcome (higher mRS scores and lower BI scores), which were consistent with the previous studies (18, 19). The patients from PSD group were also more likely to have history of coronary artery diseases and surgery which may potentially worsen the stroke patients' conditions and increase the risk of occurrence of PSD at discharge (20). Furthermore, the incidence of PSD was higher in older stroke patients, which was also consistent with the previous reports (21). This was probably because older patients were more likely to be involved in a depressive mood when dealing with the stress caused by stroke. The increased risk of PSD at discharge could also be caused by the worsening functional and cognitive impairment in the elderly (22). Previous studies have indicated that proinflammatory factors played important role in the development of PSD (23). However, there were no significant differences in proinflammatory factors between PSD and non-PSD groups which was potentially due to the different definitions of outcomes.

In recent years, the interest of psychiatrist in the association between oxidative stress and depression has grown stronger and relevant studies have shown that the depression is accompanied by increased oxidative stress and decreased antioxidant defenses (24, 25). Unlike previous studies, the present investigation focused on the occurrence of PSD at discharge and attempted to link this outcome with SUA levels on admission (15). Our study may provide an effective biomarker for psychiatrists to diagnose PSD at discharge.

Given that lower SUA had been found in major depressive and anxiety disorders (26), our study reasonably found PSD patients had lower SUA levels on admission. As a crucial central nervous system antioxidant, SUA had been proven to be an effective predictor in the development of PSD (15). Individuals with lower baseline SUA levels may be more susceptible to oxidative damage in the brain, as a result of lower antioxidant defenses and larger oxygen consumption in brain. This damage could make individuals susceptible to developing depressive mood and has also been suggested as a potential mechanism in the relapse of depression (27). As a free radical scavenger, SUA contributes to over half antioxidant capacity in plasma. As neuronal cell membranes were composed by large amounts of polyunsaturated fatty acids with a large surface area and ROS often react with lipids, optimal neuronal cell function depends on sufficient antioxidants, such as SUA, to remove ROS and protect neuronal cells from oxidative damage. Nanetti et al. found that acute ischemic stroke could cause the strong oxidative stress and generation of free radicals, and that this progression of ischemic injury could be limited by the antioxidant capacity (28). Thus, higher SUA levels could potentially protect neuron integrity and function of stroke patients and decrease the risk of PSD at discharge. Previous studies have also reported that UA has neuroprotective effects due to its potent antioxidant capacity in other diseases, such as multiple sclerosis, Alzheimer's disease, and Parkinson's disease (29–31). In acute stage of stroke, UA may exert antioxidant protection against free radical damage. Previous study has shown a negative correlation between SUA levels and stroke outcomes (32). A randomized clinical trial also showed that UA therapy was related to an improved prognosis in patients with hyperglycemia in acute stage through decreasing glucose-driven oxidative stress (33). Moreover, a phase 2 clinical trial with inosine in Parkinson's disease demonstrated a potential efficacy of increased UA on mood disorders as evaluated on the Geriatric Depression Scale. We could speculate that SUA (>480 µmol/L) may exert protective effect in PSD (34).

Moreover, UA is also a marker of purine metabolism as the final product other than antioxidant. The purinergic system has been associated with the pathophysiology of depression and is thought to influence mood, appetite, sleep, cognition, and drive through the neuromodulator adenosine and the neurotransmitter adenosine triphosphate, both of which are upstream metabolites of UA (35). Increased levels of UA are associated with decreased adenosinergic transmission and accelerated purinergic transformation, which could limit the development of depressive disorders (36). Previous studies also suggested that lower SUA levels at the onset of ischemic stroke could indicate a worse prognosis, such as increased risk of PSD, by increasing the neurological damage levels (37, 38). On the other hand, previous study also reported that the lower UA in current MDD may be related to increased exposure to ROS through mitochondrial dysfunction which could deplete UA, if not caused by lifestyle factors (39). As we all know, mitochondrial dysfunction also plays an important role in disorders of purine metabolism which could not provide sufficient UA to counteract increased oxidative stress in PSD (40).

The main strengths of this study are the sufficient sample size, well-established psychiatric diagnoses, prospective cohort study nature, and adjustment for many confounders in different models. Though further longitudinal studies are needed to confirm the association between the SUA level on admission and the risk of PSD at discharge, our study results revealed the threshold effect between them. Moreover, SUA presented affordable routine clinical biomarker which did not incur additional expense as compared with microRNAs which is limited to precise laboratory tests and not appropriate for extensive follow up and clinical application (18). Some limitations of our study should also be acknowledged. First, the stroke patients from a single center and exclusion of patients with alteration of consciousness level and severe speech disturbances may result in biases for the incidence of PSD at discharge. Second, it should be noted that the functioning of antioxidant defenses and oxidative stress is an ongoing dynamic process, the complexities of which cannot be reflected in the measurement of individual peripheral marker level at a single time point. The levels of a single biomarker of SUA may not be representative of the functioning of the whole redox homeostasis system, but nevertheless provide a clue that the system is associated with the psychopathology of PSD. Third, there were five indicators, ALB, Hcy, SUA, Hs-CRP, and hospitalization days, were collected and studied by our post-hoc analysis. The original study protocol was not designed to assess association between these five indicators and PSD risk at discharge. Given the *post hoc* nature of the analysis, the proffered mechanism would likely be speculative. Fourth, the SUA level may be influenced by metabolic profile and the information collection of glycemia, lipid profile, and metabolic syndrome were important issues (41, 42). There is no specific analysis on these issues in our study. Future longitudinal studies should further explore these associations, and SUA's potential as predictor for PSD at discharge.

In conclusion, our study suggested that lower SUA levels on admission were associated with PSD at discharge and the threshold effect was also found by RCS regression model. For stroke patients, doctors should pay attention to the baseline SUA levels for screening high risk PSD patients at discharge in clinical practice. SUA was the potential biomarker for PSD prediction. The limitation of the study includes the study biases and the results should be further confirmed in longitudinal and experimental studies.

## Data Availability Statement

The de-identified database used in the current study are available from the corresponding authors on reasonable request.

## Ethics Statement

All patients involved in this study or their family members gave written informed consents according to the Declaration of Helsinki. The approval of the study for experiments was obtained from the Ethics Committee of Tongji Medical College, Huazhong University of Science and Technology.

## Author Contributions

SZ and ZZ led the study. GL performed the data analysis and implemented the methodology. XS, WS, JM, YL, XQ, XZ, and CZ collected the data. ZZ and GL prepared the original draft. SZ reviewed and edited the final manuscript.

## Funding

This work was financially supported by the National Key R&D Program of China (grant number 2017YFC1310000), the Fundamental Research Funds for the Central Universities (grant number 2018KFYXMPT015), and Hubei Technological Innovation Special Fund (grant number 2019ACA132). The funders had no role in study design, data collection and analysis, decision to publish, or preparation of the manuscript. ZZ and SZ had full access to all the data in the study and had final responsibility for the decision to submit for publication.

## Conflict of Interest

The authors declare that the research was conducted in the absence of any commercial or financial relationships that could be construed as a potential conflict of interest.
